# Supplementary material for: Fostering participation motivation and multidisciplinary teamwork collaboration through hospital culture and team leadership in Chinese tertiary public hospitals—A cross-sectional study
Source: PLoS One. 2025 Nov 13;20(11):e0334831. doi: 10.1371/journal.pone.0334831 (PMC12614540; doi:10.1371/journal.pone.0334831)
Supplement: S1 Appendix — (DOCX) [file pone.0334831.s001.docx]

**Survey on Multidisciplinary Teamwork Behavior in Hospitals and Its Influencing Factors**

Dear participant,

This survey is conducted to understand the current status and influencing factors of multidisciplinary teamwork behaviors among healthcare professionals. The findings aim to support the improvement of patient-centered healthcare management and reforms. Please respond based on your actual situation. There are no right or wrong answers, and all your responses will remain strictly confidential. Thank you for your support!

**Section 1. Basic Information**

**1.Gender：**①Male ②Female

**2.Marital Status：**①Single ②Married ③Other

**3.Age：**①30 or below ②31~40 ③41~50 ④51~60 ⑤Over 60

**4.Professional Category：**①Clinical doctor ②Nurse ③Medical technician

**5.Professional Title:** ①Intermediate ②Associate senior ③Senior

**6.Department:** ①Internal medicine ②Surgery ③Obstetrics and gynecology④Pediatrics ⑤Medical technical department ⑥Rehabilitation ⑦Other

**7.Type of MDT you participate in:：**①Outpatient MDT ②Inpatient MDT ③Both outpatient and inpatient MDT ④Other

**Section 2. MDT Input Factors**

**Hospital Culture (Please check “✓” next to the option that best describes your agreement)**

**HC1.** Different departments within the hospital often collaborate on reform and innovation.

① Strongly disagree ② Disagree ③ Neutral ④ Agree ⑤ Strongly agree

**HC2.** Information resources within the hospital are highly shared and easily accessible.

① Strongly disagree ② Disagree ③ Neutral ④ Agree ⑤ Strongly agree

**HC3.** The hospital frequently collaborates with domestic and international peers.

① Strongly disagree ② Disagree ③ Neutral ④ Agree ⑤ Strongly agree

**HC4.** The hospital often provides support to lower-level or remote healthcare institutions.

① Strongly disagree ② Disagree ③ Neutral ④ Agree ⑤ Strongly agree

**Team Leadership (Please check “✓” next to the option that best describes your agreement)**

**TL1.** The MDT leader ensures timely discussion of all necessary cases.

① Strongly disagree ② Disagree ③ Neutral ④ Agree ⑤ Strongly agree

**TL2.** The MDT leader ensures that all members participate and contribute to discussions.

① Strongly disagree ② Disagree ③ Neutral ④ Agree ⑤ Strongly agree

**TL3.** The MDT leader can effectively resolve disagreements during case discussions.

① Strongly disagree ② Disagree ③ Neutral ④ Agree ⑤ Strongly agree

**TL4.** The MDT leader clearly assigns responsibilities for implementing treatment plans.

① Strongly disagree ② Disagree ③ Neutral ④ Agree ⑤ Strongly agree

**Section 3. MDT Process Factors**

**Participation Motivation (Please check “✓” next to the option that best describes your agreement)**

**PM1.** Participating in MDT helps improve my diagnostic and treatment skills.

① Strongly disagree ② Disagree ③ Neutral ④ Agree ⑤ Strongly agree

**PM2.** Participating in MDT helps improve my interpersonal coordination skills.

① Strongly disagree ② Disagree ③ Neutral ④ Agree ⑤ Strongly agree

**PM3.** Participating in MDT provides me with opportunities to learn from others.

① Strongly disagree ② Disagree ③ Neutral ④ Agree ⑤ Strongly agree

**PM4.** Participating in MDT brings me additional income.

① Strongly disagree ② Disagree ③ Neutral ④ Agree ⑤ Strongly agree

**Section 4. MDT Output Factors**

**MDT Behaviors (Please check “✓” next to the option that best describes your agreement)**

**MDTB1.** MDT members participate equally in the decision-making process for patient care.

① Strongly disagree ② Disagree ③ Neutral ④ Agree ⑤ Strongly agree

**MDTB2.** MDT members maintain ongoing communication to discuss treatment plans.

① Strongly disagree ② Disagree ③ Neutral ④ Agree ⑤ Strongly agree

**MDTB3.** MDT members share their successful experiences with one another.

① Strongly disagree ② Disagree ③ Neutral ④ Agree ⑤ Strongly agree

**MDTB4.** When disagreements arise, MDT members strive to find the best solutions.

① Strongly disagree ② Disagree ③ Neutral ④ Agree ⑤ Strongly agree

**MDTB5.** MDT members reflectively discuss and adjust team operations when necessary.

① Strongly disagree ② Disagree ③ Neutral ④ Agree ⑤ Strongly agree

***End of the questionnaire. Thank you for your participation.***

***We wish you all the best in your work and life!***
