# Supplementary material for: Fostering participation motivation and multidisciplinary teamwork collaboration through hospital culture and team leadership in Chinese tertiary public hospitals—A cross-sectional study
Source: PLoS One. 2025 Nov 13;20(11):e0334831. doi: 10.1371/journal.pone.0334831 (PMC12614540; doi:10.1371/journal.pone.0334831)
Supplement: Attachment — Submitted filename: Reviewed PONE-D-25-03210.pdf [file pone.0334831.s003.pdf]

PLOS ONE

Fostering participation motivation and multidisciplinary teamwork collaboration through hospital culture and team leadership in Chinese tertiary public hospitals

--Manuscript Draft--

Manuscript Number:	PONE-D-25-03210
Article Type:	Research Article
Full Title:	Fostering participation motivation and multidisciplinary teamwork collaboration through hospital culture and team leadership in Chinese tertiary public hospitals
Short Title:	Fostering motivation and collaboration through hospital culture and team leadership
Corresponding Author:	xianhong Huang<br>Hangzhou Normal University<br>hangzhou, CHINA
Keywords:	Hospital Culture; Team Leadership; Participation Motivation; Multidisciplinary Teamwork Behavior
Abstract:	Many modern diseases require more than one discipline for effective treatment, making multidisciplinary teamwork (MDT) essential. However, research on MDT in tertiary public hospitals in China is limited. These large healthcare institutions require effective collaboration among various departments to manage complex cases. Therefore, this study examined the effects of hospital culture, team leadership, and participation motivation on the MDT behavior of healthcare professionals to enhance MDT and improve related services. We conducted a questionnaire survey of 425 multidisciplinary team members in tertiary public hospitals in Hangzhou. T-tests, analysis of variance, and hierarchical linear regression were used to analyze the state of healthcare professionals' MDT behaviors and the associated factors. A path analysis with Structural Equation Model was used to explore and verify the effects of hospital culture, team leadership, and participation motivation on MDT behavior, as well as their mechanism. The findings revealed significant positive effects of hospital culture, team leadership, and participation motivation on MDT behavior. An SEM path analysis confirmed that these factors directly influence MDT behavior. Moreover, hospital culture and team leadership indirectly affected MDT behavior through participation motivation. This study demonstrated that a positive hospital culture and team leadership significantly enhanced MDT, with participation motivation mediating this relationship. These findings suggest that hospital leadership should promote a proactive and harmonious hospital culture and facilitate the development of team leaders' management skills. Furthermore, exploring diverse incentives to increase healthcare professionals' participation motivation is essential for advancing MDT.
Order of Authors:	Zhengnan Meng
	Jiamin Tang
	Jie Yang
	Huaineng Wu
	Linlin Cui
	Xiaohe Wang
	xianhong Huang
Additional Information:	
Question	Response
**Financial Disclosure**<br><br>Enter a financial disclosure statement that describes the sources of funding for the work included in this submission. Review	Yes

Powered by Editorial Manager® and ProduXion Manager® from Aries Systems Corporation

the submission guidelines for detailed requirements. View published research articles from *PLOS ONE* for specific examples.<br><br>This statement is required for submission and **will appear in the published article** if the submission is accepted. Please make sure it is accurate.<br><br>**Funded studies**<br>Enter a statement with the following details:<br>• Initials of the authors who received each award<br>• Grant numbers awarded to each author<br>• The full name of each funder<br>• URL of each funder website<br>• Did the sponsors or funders play any role in the study design, data collection and analysis, decision to publish, or preparation of the manuscript?<br><br>Did you receive funding for this work?	
Please add funding details.<br>as follow-up to "**Financial Disclosure**<br><br>Enter a financial disclosure statement that describes the sources of funding for the work included in this submission. Review the submission guidelines for detailed requirements. View published research articles from *PLOS ONE* for specific examples.<br><br>This statement is required for submission and **will appear in the published article** if the submission is accepted. Please make sure it is accurate.	This research was funded by the National Natural Science Foundation of China Project (grant numbers 72274051 and 71974050) and Hangzhou Medicine and Health Science and Technology Program(grant number ZD20220099).

**Funded studies**<br>Enter a statement with the following details:<br>• Initials of the authors who received each award<br>• Grant numbers awarded to each author<br>• The full name of each funder<br>• URL of each funder website<br>• Did the sponsors or funders play any role in the study design, data collection and analysis, decision to publish, or preparation of the manuscript?<br><br>Did you receive funding for this work?"	
Please select the country of your main research funder (please select carefully as in some cases this is used in fee calculation).<br>as follow-up to "**Financial Disclosure**<br><br>Enter a financial disclosure statement that describes the sources of funding for the work included in this submission. Review the submission guidelines for detailed requirements. View published research articles from *PLOS ONE* for specific examples.<br><br>This statement is required for submission and **will appear in the published article** if the submission is accepted. Please make sure it is accurate.<br><br>**Funded studies**<br>Enter a statement with the following details:<br>• Initials of the authors who received each award<br>• Grant numbers awarded to each author<br>• The full name of each funder<br>• URL of each funder website<br>• Did the sponsors or funders play any role in the study design, data collection and analysis, decision to publish, or preparation of the manuscript?<br><br>Did you receive funding for this work?"	CHINA - CN
**Competing Interests**	The authors declare no competing interests.

Use the instructions below to enter a competing interest statement for this submission. On behalf of all authors, disclose any competing interests that could be perceived to bias this work—acknowledging all financial support and any other relevant financial or non-financial competing interests.<br><br>This statement is required for submission and **will appear in the published article** if the submission is accepted. Please make sure it is accurate and that any funding sources listed in your Funding Information later in the submission form are also declared in your Financial Disclosure statement.<br><br>View published research articles from *PLOS ONE* for specific examples.<br><br>**NO authors have competing interests**<br><br>Enter: *The authors have declared that no competing interests exist.*<br><br>**Authors with competing interests**<br><br>Enter competing interest details beginning with this statement:<br><br>*I have read the journal's policy and the authors of this manuscript have the following competing interests: [insert competing interests here]*<br><br>* typeset	
**Ethics Statement**<br><br>Enter an ethics statement for this submission. This statement is required if the study involved:<br><br>• Human participants<br>• Human specimens or tissue<br>• Vertebrate animals or cephalopods<br>• Vertebrate embryos or tissues	This study was reviewed and approved on March 3rd, 2022, by the Research Ethics Committee of Hangzhou Normal University, including the study protocol, informed consent form, and questionnaire (Approval Number: 2022-1121). The research adhered to the ethical guidelines outlined in the 1964 Declaration of Helsinki and its subsequent amendments. Each participant provided informed consent prior to participation.

• Field research<br><br>Write "N/A" if the submission does not require an ethics statement.<br><br>General guidance is provided below. Consult the submission guidelines for detailed instructions. **Make sure that all information entered here is included in the Methods section of the manuscript.**	

**Format for specific study types**<br><br>**Human Subject Research (involving human participants and/or tissue)**<br>• Give the name of the institutional review board or ethics committee that approved the study<br>• Include the approval number and/or a statement indicating approval of this research<br>• Indicate the form of consent obtained (written/oral) or the reason that consent was not obtained (e.g. the data were analyzed anonymously)<br><br>**Animal Research (involving vertebrate animals, embryos or tissues)**<br>• Provide the name of the Institutional Animal Care and Use Committee (IACUC) or other relevant ethics board that reviewed the study protocol, and indicate whether they approved this research or granted a formal waiver of ethical approval<br>• Include an approval number if one was obtained<br>• If the study involved *non-human primates*, add *additional details* about animal welfare and steps taken to ameliorate suffering<br>• If anesthesia, euthanasia, or any kind of animal sacrifice is part of the study, include briefly which substances and/or methods were applied<br><br>**Field Research**<br><br>Include the following details if this study involves the collection of plant, animal, or other materials from a natural setting:<br>• Field permit number<br>• Name of the institution or relevant body that granted permission	
**Data Availability**<br><br>Authors are required to make all data underlying the findings described fully available, without restriction, and from the time of publication. PLOS allows rare exceptions to address legal and ethical concerns. See the PLOS Data Policy and FAQ for detailed information.	No - some restrictions will apply

A Data Availability Statement describing where the data can be found is required at submission. Your answers to this question constitute the Data Availability Statement and **will be published in the article**, if accepted.<br><br>**Important:** Stating 'data available on request from the author' is not sufficient. If your data are only available upon request, select 'No' for the first question and explain your exceptional situation in the text box.<br><br>Do the authors confirm that all data underlying the findings described in their manuscript are fully available without restriction?	
**Describe where the data may be found in full sentences. If you are copying our sample text, replace any instances of XXX with the appropriate details.**<br><br>• If the data are **held or will be held in a public repository**, include URLs, accession numbers or DOIs. If this information will only be available after acceptance, indicate this by ticking the box below. For example: *All XXX files are available from the XXX database (accession number(s) XXX, XXX.*).<br>• If the data are all contained **within the manuscript and/or Supporting Information files**, enter the following: *All relevant data are within the manuscript and its Supporting Information files.*<br>• If neither of these applies but you are able to provide **details of access elsewhere**, with or without limitations, please do so. For example:<br><br>*Data cannot be shared publicly because of [XXX]. Data are available from the XXX Institutional Data Access / Ethics Committee (contact via XXX) for researchers who meet the criteria for access to confidential data.*<br><br>*The data underlying the results presented in the study are available from (include the name of the third party*	The datasets generated during and/or analyzed during the current study are available from the corresponding author upon reasonable request.

and contact information or URL).<br>• This text is appropriate if the data are owned by a third party and authors do not have permission to share the data.<br><br>* typeset	
Additional data availability information:	

Manuscript

Click here to access/download;Manuscript;manuscript(2025.01.19).docx

Fostering participation motivation and multidisciplinary teamwork collaboration through hospital culture and team leadership in Chinese tertiary public hospitals

Short title: Fostering motivation and collaboration through hospital culture and team leadership

Zhengnan Meng[1], Jiamin Tang[1], Jie Yang[1], Huaineng Wu[2], Linlin Cui[3], Simin Zhu[2], Xiaohe Wang[1], and Xianhong Huang[1]

[1] School of Public Health, Hangzhou Normal University, Hangzhou, China

[2] Affiliated Mental Health Center and Hangzhou Seventh People's Hospital, Zhejiang University School of Medicine, China

[3] Hangzhou International Urbanology Research Center and Center for Urban Governance Studies, China

Abstract

Many modern diseases require more than one discipline for effective treatment, making multidisciplinary teamwork (MDT) essential. However, research on MDT in tertiary public hospitals in China is limited. These large healthcare institutions require effective collaboration among various departments to manage complex cases. Therefore, this study examined the effects of hospital culture, team leadership, and participation motivation on the MDT behavior of healthcare professionals to enhance MDT and improve related services. We conducted a questionnaire survey of 425 multidisciplinary team members in tertiary public hospitals in Hangzhou. T-tests, analysis of variance, and hierarchical linear regression were used to analyze the state of healthcare professionals' MDT behaviors and the associated factors. A path analysis with Structural Equation Model was used to explore and verify the effects of hospital culture, team leadership, and participation motivation on MDT behavior, as well as their mechanism. The findings revealed significant positive effects of hospital culture, team leadership, and participation motivation on MDT behavior. An SEM path analysis confirmed that these factors directly influence MDT behavior. Moreover, hospital culture and team leadership indirectly affected MDT behavior through participation motivation. This study demonstrated that a positive hospital culture and team leadership significantly enhanced MDT, with participation motivation mediating this relationship. These findings suggest that hospital leadership should promote a proactive and harmonious hospital culture and facilitate the development of team leaders' management skills. Furthermore, exploring diverse incentives to increase healthcare professionals' participation motivation is essential for advancing MDT.

Keywords: Hospital Culture; Team Leadership; Participation Motivation; Multidisciplinary Teamwork Behavior

1. Introduction

The medical treatment model is continuously evolving. Traditionally, the diagnosis and treatment of diseases have depended on a single-discipline approach. However, recent studies have shown that this method fails to address patients' comprehensive needs [1,2]. As society advances, the medical model has transitioned to a bio-psycho-social approach, prompting healthcare professionals to consider the whole patient and address complex issues using integrated medical knowledge [3]. To enhance the quality of care, healthcare institutions are increasingly adopting multidisciplinary teamwork (MDT). This model brings together experts from various disciplines to form a regular team that discusses and provides comprehensive treatment recommendations [4]. This allows healthcare professionals to analyze issues from multiple perspectives and deliver effective patient care. Research indicates that MDT can improve patient survival rates [5], increase satisfaction with healthcare services [6], and enhance clinicians' diagnostic skills [7] and job satisfaction [8]. However, challenges, such as inadequate inter-departmental teamwork [4] and low engagement of healthcare professionals in MDT [9], persist, preventing the full realization of the MDT model in medical practice. In China, approximately 70% of adverse events result from teamwork failures [10], significantly wasting healthcare resources [11]. Therefore, identifying the factors that influence MDT in tertiary public hospitals and their underlying mechanisms is essential to promote the use of MDT in clinical practice, enhance healthcare quality, and facilitate disease treatment development in public hospitals.

Research on factors influencing MDT has largely concentrated on internal organizational elements, such as hospital culture, team leadership, participation motivation, and organizational climate [12-14]. However, existing studies have notable

gaps. First, few studies have examined the cooperative behaviors of MDT healthcare professionals as an outcome variable, despite the fact that understanding these behaviors is essential because healthcare professionals are the pillar of MDT. Second, although hospital culture, team leadership, and participation motivation are critical factors impacting MDT, comprehensive research on their pathways of influence remains insufficient. Furthermore, hospital culture and team leadership influence participation motivation [15,16]. However, studies examining the mediating role of participation motivation in the effects of hospital culture and team leadership on MDT are lacking. Therefore, this study investigated the impact of hospital culture, team leadership, and participation motivation on MDT behavior.

To address the limitations of previous studies, this study adopted the Input-Process-Output (IPO) model as a theoretical framework to analyze the influencing paths affecting MDT. The IPO model, a well-established framework for examining team collaboration, states that team inputs directly impact outputs and indirectly influence team processes [17]. Inputs encompass various factors that influence team members, such as individual characteristics (e.g., personality and skills), team dynamics (e.g., structure and cohesion), and external conditions (e.g., organizational climate and support). Processes pertain to the behaviors team members engage in to achieve their goals, whereas outputs reflect team performance and individual emotional responses, such as satisfaction and commitment [18]. This model has frequently been used in healthcare research to examine the direct effects of input factors, such as knowledge, skills, and organizational support, on output factors, such as team member satisfaction and effectiveness, as well as their indirect effects through process factors such as interpersonal relationships, communication skills, and team reflexivity [19,20]. MDT behaviors in tertiary public hospitals are shaped by input factors, such as hospital

culture, team leadership, and structure, as well as process factors, such as participation motivation and interaction intention among members.

This study focused on healthcare professionals engaged in MDT in tertiary public hospitals in Hangzhou, China. This study used the IPO model to analyze the effects of hospital culture, team leadership, and participation motivation on MDT behavior, including the mediating role of participation motivation. This empirical study aimed to elucidate the complex relationships between input and output factors and develop a model outlining factors influencing MDT behavior. A structural equation analysis was used to overcome the limitations of previous research regarding the factors and pathways affecting MDT behavior. This study sought to improve the quality of medical diagnosis and treatment and provide a theoretical framework for policymakers to guide the development of MDT in tertiary public hospitals.

2. Literature review and hypothesis development

2.1 The influence of hospital culture on MDT behavior

Hospital culture encompasses values, beliefs, and attitudes that connect healthcare professionals [21]. Studies have shown that hospital culture positively influences teamwork. A longitudinal study in Norwegian surgical wards revealed that collaborative cultural environments fostered trust among team members, promoting participation in teamwork [22]. Qin et al. [23] examined the missions, visions, and values of five leading hospitals and found that a patient-centered culture significantly enhanced collaboration among healthcare professionals. Moreover, a hospital culture that prioritizes employee satisfaction, team safety [24], effective communication and mutual collaboration [25], and innovation significantly enhances teamwork among healthcare professionals across different departments [26]. Thus, existing research suggests that the core values and ethical standards of hospital culture positively

influence the MDT behaviors of healthcare professionals by fostering a supportive work environment. Therefore, we proposed the following hypothesis:

H1: Hospital culture positively affects MDT behavior.

2.2 The influence of team leadership on MDT behavior

Team leadership encompasses a leader's ability to effectively communicate the team's mission and objectives while guiding and influencing members toward achieving these goals [27]. Research shows that the leadership skills of the individual overseeing MDT are crucial for fostering cooperation among team members [28]. Beiboer et al. [29] found that strong team leadership enhanced constructive collaboration in clinical settings. Akinsola et al. [30] demonstrated that effective team leadership facilitates communication among individuals, resolves issues, and promotes teamwork. Moreover, a study examining teamwork among oncology nursing teams identified team leadership as a key factor influencing nurses' teamwork behavior [31]. Tremblay et al. [32] evaluated the collaborative conditions in oncology MDT and revealed that professional attributes, resource allocation, policy support, and team leadership were vital for effective team functioning. This suggests that the leadership exhibited by team leaders in areas such as goal setting and conflict resolution can positively impact MDT among healthcare professionals. Therefore, we proposed the following hypothesis:

H2: Team leadership positively affects MDT behavior.

2.3 The influence and mediating roles of participation motivation

Participation motivation is an internal drive fueled by the desire to achieve specific goals [33]. Healthcare professionals' participation motivation is essential for delivering high-quality healthcare services and fostering effective teamwork [34].Yahang et al. examined how participation motivation indirectly improves team performance by

influencing teamwork behavior [35]. The motivation of healthcare professionals to engage in their work significantly impacts their MDT behavior. Research [36] indicates that the integration and reform of the healthcare service system can boost healthcare professionals' motivation, thereby enhancing their enthusiasm for teamwork.

Furthermore, participation motivation is a key factor linking hospital culture and MDT behavior. Chmielewska et al. [37] discovered that a hospital culture characterized by harmonious interaction enhanced healthcare professionals' organizational commitment and participation motivation, improving overall hospital performance. Huang et al. [38] analyzed the effects of hospital culture on patient-centered care and reported that effective internal communication and a culture of innovation significantly boosted healthcare professionals' motivation to engage in their work, thereby facilitating cross-team collaboration.

Moreover, studies have shown that participation motivation is crucial in linking team leadership and MDT behavior. A survey conducted in various regions of Uganda revealed that leadership styles were significantly associated with job satisfaction, participation motivation, and teamwork among healthcare professionals [39]. Subramaniam et al. [40] demonstrated that participation motivation mediated the relationship between leadership and safety behavior among nurses. Chaman et al. [41] found that employees' internal participation motivation mediated the relationship between team leadership style and knowledge sharing, which was essential for fostering organizational cooperation. Tran and Hoang [42] discovered that transformational leadership positively influenced job performance, with employee participation serving as a mediating factor.

These findings indicate that hospital culture, team leadership, participation motivation, and MDT behavior are interrelated. Furthermore, participation motivation

mediates the relationship between various antecedent variables, such as team leadership, hospital culture, social support, and professional resources, and outcome variables, such as cooperative behavior, job satisfaction, turnover intention, and job performance. Therefore, we proposed the following hypotheses:

H3: Participation motivation positively affects MDT behavior.

H4a: Participation motivation mediates the relationship between hospital culture and MDT behavior.

H4b: Participation motivation mediates the relationship between team leadership and MDT behavior.

The theoretical model of this study is presented in Fig 1.

Fig 1. Theoretical model of the relationships between hospital culture, team leadership, and MDT behavior of healthcare professionals in tertiary public hospitals.

3. Methods

3.1 Participants and procedures

With its rich medical resources, Hangzhou has become a leading city in implementing MDT models in tertiary hospitals. Therefore, this study focused on tertiary public hospitals in Hangzhou and used purposive sampling to survey doctors, nurses, and medical technicians engaged in MDT collaboration.

Participants were chosen from tertiary public hospitals in Hangzhou that have adopted an MDT model for diagnosis and treatment. The inclusion criteria were (1) being a clinical doctor, nurse, or medical technician engaged in MDT and (2) being currently employed and having participated in MDT for more than six months during the survey. The exclusion criteria were (1) being a healthcare professional visiting for training or education; (2) being a healthcare professional currently off duty; (3) being a

healthcare professional who only attend MDT case discussions as listener and does not have any experience participating in MDT; and (4) being administrative or support personnel involved in MDT organization and management. From June 10, 2022, to July 20, 2023, We distributed 500 questionnaires and obtained 452 valid responses, which were collected on-site, yielding a valid response rate of 90.4%.

3.2 Measures

A general survey form was used to collect essential demographic and occupational information from healthcare professionals involved in MDT. The form included six items: gender (men, women), age (≤ 30 years, 31–40 years, 41–50 years, 51–60 years, ≥ 61 years), professional category (clinical doctor, nurse, medical technician), professional title (intermediate, associate senior, full senior), department (internal medicine, surgery, obstetrics and gynecology, pediatrics, medical technology, rehabilitation, other), and type of MDT participation (outpatient, inpatient, both, other).

Hospital culture was measured using a four-item unidimensional scale adapted from The Hospital Culture Questionnaire developed by Körner et al. [43]. Responses were rated on a five-point Likert scale (1 = highly inconsistent; 5 = highly consistent), with higher scores indicating a more favorable hospital culture. The scale demonstrated strong reliability in this study, with a Cronbach's alpha coefficient of 0.829, composite reliability (CR) value of 0.81, factor loadings between 0.56 and 0.84, and average variance extracted (AVE) value of 0.52.

Leadership was measured using a four-item unidimensional scale adapted from 'A Tumor Leadership Assessment instrument' (ATLAS) developed by Jalil et al. [44]. Responses were rated on a five-point Likert scale (1 = highly inconsistent; 5 = highly consistent), with higher scores indicating stronger team leadership. The scale demonstrated strong reliability and validity in this study, with a Cronbach's alpha

coefficient of 0.913, CR value of 0.91, factor loadings between 0.80 and 0.87, and AVE value of 0.73.

Participation motivation was measured using a four-item unidimensional scale adapted from The Work Extrinsic and Intrinsic Motivation Scale (WEIMS) developed by Tremblay et al. [45]. Responses were rated on a five-point Likert scale (1 = highly inconsistent; 5 = highly consistent), with higher scores indicating higher participation motivation. The scale demonstrated good reliability and validity in this study, with a Cronbach's alpha coefficient of 0.739, CR value of 0.84, factor loadings ranging from 0.72 to 0.85, and AVE value of 0.64.

MDT behavior was measured using a five-item simplified version of the Interprofessional Teamwork Scale, which was translated and adapted by Jin et al. [46]. Responses were rated on a five-point Likert scale (1 = highly inconsistent; 5 = highly consistent), with higher scores indicating more efficient MDT behavior. The scale demonstrated strong reliability and validity in this study, with a Cronbach's alpha coefficient of 0.882, CR value of 0.89, factor loadings between 0.72 and 0.85, and AVE value of 0.61.

3.3 Ethical considerations

This study was reviewed and approved on March 3rd, 2022, by the Research Ethics Committee of Hangzhou Normal University, including the study protocol, informed consent form, and questionnaire (Approval Number: 2022-1121). The research adhered to the ethical guidelines outlined in the 1964 Declaration of Helsinki and its subsequent amendments. Each participant provided informed consent prior to participation.

3.4 Statistical analysis

The data analysis was performed using SPSS 27.0 and Amos 25.0. The reliability of the questionnaire was evaluated using CR values and internal consistency

(Cronbach's alpha coefficient), whereas structural validity was assessed through a confirmatory factor analysis. Content validity was determined using the item-to-total correlation method. Descriptive statistics were used to outline the demographic and occupational characteristics of the participants, along with the current state of hospital culture and team leadership. Categorical variables were reported as frequencies and proportions, whereas continuous variables with a normal distribution were summarized using the mean and standard deviation. Univariate analysis was used to compare participation motivation and MDT behavior scores among MDT members with varying demographic and occupational characteristics. For normally distributed variables, t-tests were employed for comparisons between two groups, whereas a one-way analysis of variance (ANOVA) was used for comparisons involving three or more groups. A hierarchical linear regression analysis using the Enter method was applied to identify the primary factors influencing healthcare professionals' participation in MDT behavior in tertiary public hospitals.

A structural equation model was developed using Amos 25.0 to examine the direct and indirect effects of hospital culture, team leadership, and participation motivation on MDT behavior. The bootstrap method was used to test the mediating effect of participation motivation. The model comprised two primary pathways: hospital culture influencing participation motivation, which affected cooperative behavior; and team leadership impacting participation motivation, which affected cooperative behavior. The model fit was evaluated and adjusted based on modification indices.

4. Results

4.1 Participant characteristics

The participants' demographic characteristics are presented in Table 1.

Table 1. Participants characteristic (N = 452)

Variable	Category	Frequency	Percentage (%)
Gender	Male	246	54.4
	Female	206	45.6
Age (years)	⩽ 30	15	3.3
	31–40	176	38.9
	41–50	193	42.7
	⩾ 51	68	15.1
Department	Internal medicine	168	37.2
	Surgery	115	25.4
	Obstetrics and gynecology	15	3.3
	Pediatrics	30	6.6
	Medical technology	98	21.7
Professional Category	Clinical doctor	357	79.0
	Nurses and medical technicians	95	21.0
Professional title	Intermediate	116	25.7
	Associate senior	193	42.7
	Full senior	143	31.6
MDT participation type	Outpatient	14	3.1

Inpatient	253	56.0
Outpatient and inpatient	174	38.5
Other	11	2.4

4.2 Correlations between hospital culture, team leadership, participation motivation, and MDT behavior

As shown in Table 2, hospital culture was positively correlated with team leadership, participation motivation, and MDT behavior. Team leadership was positively correlated with participation motivation and MDT behavior. Furthermore, participation motivation was positively correlated with MDT behavior.

Table 2. Correlations between hospital culture, team leadership, participation motivation, and MDT behavior

**Variable**	**Mean±SD**	**Hospital culture**	**Team leadership**	**Participation motivation**	**MDT behavior**
**Hospital culture**	3.74±0.62	1			
**Team leadership**	4.13±0.56	0.540**	1		
**Participation motivation**	3.80±0.56	0.544**	0.541**	1	
**MDT behavior**	3.98±0.56	0.490**	0.651**	0.554**	1

** $P < 0.01$ (two-tailed).

4.3 Hierarchical linear regression results for MDT behavior

First, unordered categorical data that demonstrated statistical significance in the univariate analysis of healthcare professionals' cooperative behavior, such as professional category, title, and type of MDT participation, were dummy-coded. Hierarchical linear regression was conducted with healthcare professionals' cooperative behavior as the dependent variable. The independent variables were categorized into four layers: the first layer comprised demographic variables, the second layer added hospital culture variables, the third layer added team leadership variables, and the fourth layer added participation motivation variables. The results indicated that incorporating demographic characteristics, hospital culture, team leadership, and participation motivation in the regression model produced statistically significant changes in ΔR^2. Analyzing the changes in ΔR^2 values revealed that hospital culture exerted a more substantial influence on healthcare professionals' MDT behavior than demographic characteristics, team leadership, and participation motivation, explaining 23.3% of the variance in this behavior.

As shown in Table 3, an analysis of the independent variables in the fourth-layer model revealed that among healthcare professionals holding the same highest title, those with an intermediate title exhibited the highest scores for cooperative behavior. Hospital culture positively influenced the MDT behavior of healthcare professionals ($\beta = 0.126$, $P = 0.004$). Moreover, team leadership significantly enhanced MDT behavior ($\beta = 0.443$, $P < 0.001$), and participation motivation further increased MDT behavior ($\beta = 0.252$, P < 0.001).

Table 3. Hierarchical regression results for MDT behavior

Variable	First block	Second block	Third block	Fourth block
	Standardized β	Standardized β	Standardized β	Standardized β
Post (reference: clinical doctor)				
Nurse	0.047	0.015	0.010	0.022
Medical technician	-0.087	-0.016	-0.006	-0.007
Professional title (reference: full senior)				
Intermediate	-0.057	-0.095*	-0.142**	-0.142**
Associate senior	-0.171*	-0.195**	-0.201**	-0.197**
MDT participation type (reference: outpatient and inpatient)				
Outpatient	-0.107*	-0.043	-0.012	-0.013
Inpatient	-0.031	0.050*	0.072	0.067
Other	-0.125*	-0.091*	-0.026	-0.045
Hospital culture		0.506**	0.217**	0.126*
Team leadership			0.533**	0.443**
Participation Motivation				0.252**
R^2	0.062	0.295	0.488	0.527
F	4.193**	23.216**	46.836**	49.118**
$\triangle R^2$	0.062	0.233	0.193	0.193
$\triangle F$	4.193**	146.744**	166.432**	36.144**
VIF_{max}	1.446	1.452	1.526	1.739

$*P < 0.05$, $** P < 0.001$. VIF_{max}, variance inflation factor maximum.

4.4 Structural equation model results for MDT behavior

4.4.1 Model construction

A structural equation model was developed using hospital culture, team leadership, participation motivation, and MDT behavior as latent variables. The specific dimensions of hospital culture, team leadership, participation motivation, and MDT behavior served as observed variables (Fig 2). The model fit results revealed a χ^2/df ratio of 3.137, with all goodness-of-fit indices (GFI, NFI, CFI, TLI) exceeding 0.9. RMSEA was below 0.08, and AGFI was above 0.8, indicating a good model fit.

Fig 2. Model of the influencing mechanism of MDT behavior among healthcare professionals.

Table 4. Structural equation model fit results

Fit indices	Standards	Model fit
**χ^2/df**	3–5 acceptable; 1–3 good	3.137
**GFI**	> 0.8 acceptable; > 0.9 good	0.922
**AGFI**	> 0.8 acceptable; > 0.9 good	0.891
**RMSEA**	< 0.1 acceptable; < 0.08 good	0.069
**IFI**	> 0.8 acceptable; > 0.9 good	0.954
**CFI**	> 0.8 acceptable; > 0.9 good	0.954
**NFI**	> 0.8 acceptable; > 0.9 good	0.934
**TLI**	> 0.8 acceptable; > 0.9 good	0.943

4.4.2 Impact pathways of MDT behavior

As shown in Table 5, the standardized effect analysis results indicated that the culture of tertiary public hospitals and team leadership significantly enhanced MDT behavior. Furthermore, hospital culture and team leadership directly influenced MDT

behavior and had indirect effects through participation motivation, supporting H1 and H2. Moreover, participation motivation significantly and directly affected MDT behavior, supporting H3.

Table 5. Influencing path coefficients on MDT behavior

**Relationship between variables**	**Standardized direct effect**	**Standardized indirect effect**	**Standardized total effect**	$\boldsymbol{P}$	**Supported hypothesis**
**Hospital culture → Participation motivation → MDT behavior**	0.180	0.062	0.242	$P = 0.001$	H1
**Team leadership → Participation motivation → MDT behavior**	0.393	0.171	0.564	$P < 0.001$	H2
**Participation motivation → MDT behavior**	0.335	/	0.335	$P < 0.001$	H3

4.4.3 Mediation effects of participation motivation

The mediation effect of participation motivation on MDT behavior among healthcare professionals was assessed using the bootstrap method, with a sample size of 2000 and a 95% confidence interval. The analysis used the maximum likelihood estimation approach. The results revealed total effect values of 0.242 for hospital culture and 0.564 for team leadership on MDT behavior. The confidence interval analysis did not include zero, indicating that the overall mediation effect was significant. Hospital culture and team leadership directly affect MDT behavior with effect values of 0.180 and 0.393, respectively, and confidence intervals not including 0, indicating significant direct effects. Additionally, hospital culture and team leadership also indirectly affect MDT behavior, with effect values of 0.062 and 0.171, respectively, and confidence intervals not including 0, indicating significant indirect effects. Thus, both the direct and indirect effects of hospital culture and team leadership on MDT behavior were significant, indicating partial mediation, which supported H4 (Table 6).

Table 6. Mediation effect bootstrap analysis results (standardized coefficients)

Path	Effect type	S.E.	Effect size	Bias-corrected 95%CI			Percentile 95%CI		
				Lower	Upper	*P*	Lower	Upper	*P*
Hospital culture → Participation motivation → MDT behavior	Total	0.054	0.242	0.138	0.352	0.001	0.133	0.346	0.001
	Direct	0.051	0.180	0.079	0.280	0.001	0.075	0.277	0.001
	Indirect	0.024	0.062	0.022	0.114	0.004	0.020	0.112	0.005
Team leadership → Participation motivation → MDT behavior	Total	0.063	0.564	0.436	0.680	0.001	0.444	0.685	0.001
	Direct	0.068	0.393	0.258	0.524	0.001	0.260	0.526	0.001
	Indirect	0.043	0.171	0.101	0.274	0.001	0.096	0.266	0.001

5. Discussion

This study used the IPO model and developed a framework to explore the influencing pathways of MDT behavior among healthcare professionals. It systematically analyzed the effects of the culture of tertiary public hospitals, team leadership, and healthcare professionals' motivation to participate in this behavior. The results indicated that hospital culture, team leadership, and participation motivation significantly influenced MDT behavior. In particular, a positive hospital culture and effective team leadership enhanced healthcare professionals' motivation to participate, which in turn fostered the development of MDT behavior. Moreover, participation motivation mediated the relationships between hospital culture, team leadership, and MDT behavior. This suggests that when healthcare professionals are highly motivated to participate, the effects of hospital culture and team leadership on their teamwork behavior are amplified.

5.1 Current status of MDT among healthcare professionals

The research findings showed an average score of 3.98±0.56 for MDT among healthcare professionals, indicating that while the implementation in tertiary public hospitals in Hangzhou was generally effective, it had potential for improvement. This score was lower than that reported by Lusi et al. [47], which could be attributed to differences in the research participants. Lusi et al. [47] focused on nursing teams in tertiary hospitals, where members had long-standing relationships and a strong understanding of each other, facilitating collaboration. In contrast, MDT is a relatively new diagnostic and treatment model that is being piloted in many hospitals, indicating that team dynamics require further development. Furthermore, the values in this study were lower than those reported by Yutong et al. [48], who examined the teamwork abilities of nursing graduate students. Their focus on disciplines with high professional

compatibility likely facilitated teamwork, as it did not involve complex and challenging medical cases. Thus, while the MDT behavior values in this study were slightly low, the promotion and implementation of MDT in China are evolving. Thus, healthcare institutions should improve collaboration among MDT members through targeted training and structural enhancements while developing relevant policies to strengthen MDT models and enhance MDT effectiveness.

5.2 The influence of hospital culture on MDT behavior

The hierarchical linear regression results indicated that hospital culture substantially influenced MDT behavior more than demographic characteristics, team leadership, and participation motivation. This aligned with the findings of Pavithra and Westbrook [49]. A positive hospital culture fosters the coordination and communication skills of healthcare professionals, thereby enhancing MDT. DiazGranados et al. [50] found that the unique culture of hospitals shaped the interactions among team members. Moreover, a systematic review revealed that hospital culture influenced the collaborative atmosphere among MDT members, ultimately impacting team collaboration [51].

Furthermore, supportive policies and a strong cooperative culture in hospitals enhance trust among team members, leading to positive collaborative interactions [14]. Safety culture, which is an integral aspect of hospital organizational culture, is vital for ensuring patient safety. Implementing safety culture training can positively influence MDT behavior, contributing to the formation of high-performance healthcare teams [52]. Thus, health administration departments and hospital managers should enhance hospital culture, cultivate a positive and harmonious work environment, encourage communication and teamwork among healthcare professionals, and support the team's sustainable development. In addition, hospitals should consider improving healthcare

professionals' coordination and communication skills through regular training and cultural development initiatives.

5.3 The influence of team leadership on MDT behavior

This study revealed a positive relationship between team leadership and collaborative behaviors in MDT. This finding aligns with that of Chao et al. [53], who found that MDT leaders could enhance healthcare professionals' participation by using leadership skills such as effective task allocation, quick decision-making, and fostering open discussions among team members. Furthermore, a mature leadership approach fosters a democratic environment, encourages meaningful discussions, and enhances MDT [54]. Strong team leadership typically improves the overall capabilities of the team, encourages active participation from members, and supports effective MDT. Thus, hospital administration should prioritize the development of team leadership and enhance leaders' coordination and decision-making skills through targeted initiatives. Suggested initiatives include regular leadership training focusing on team coordination, decision-making, and communication skills; implementing a feedback system to encourage self-reflection and ongoing development for MDT leaders; and enhancing leadership capabilities to advance MDT.

5.4 The influence of participation motivation on MDT behavior

This study identified a positive relationship between healthcare professionals' motivation to participate and engagement in MDT, which aligned with previous study findings [55]. This implies that proactive involvement of team members is essential for the successful functioning of MDT. Members who are highly motivated to participate in MDT tend to adopt a patient-centered approach, which enhances their ability to communicate effectively with patients and improves their overall medical experience. According to Latham et al. [56], when employees have relevant knowledge and are

open to sharing it, their motivation to engage in their work increases, fostering mutual learning and collaboration and enhancing team performance. Moreover, Karaferis et al. [57] found that when doctors were provided opportunities to engage in decision-making relevant to their roles, they became invested in their work and developed collaborative relationships with their peers. Thus, hospital administration should proactively implement reward systems, career development initiatives, and enhancements to the work environment to boost healthcare professionals' motivation to engage, thereby fostering MDT.

5.5 The mediating role of participation motivation in the relationship between hospital culture and MDT behavior

The bootstrap analysis indicated that participation motivation partially mediated the influence of hospital culture on collaborative behavior in MDT. This aligns with previous study findings indicating that fostering a positive hospital culture enhances healthcare professionals' motivation to engage in their work, encourages collaboration, and ultimately improves patient outcomes [15]. Furthermore, McFadden et al. [58] showed that a hospital culture that prioritizes teamwork, respect, and fairness increased healthcare professionals' motivation to participate, leading to more frequent collaboration among teams. This study revealed that a positive and proactive culture in tertiary public hospitals was correlated with increased work motivation among healthcare professionals and more frequent MDT. Thus, hospital culture and team leadership serve as input factors that directly influence multidisciplinary collaboration while impacting it indirectly through the mediation of participation motivation. Positive participation motivation is a critical link between hospital culture and collaborative behavior in MDT. These findings support the IPO model, which asserts that team inputs influence outcomes directly and through team processes [18]. Therefore, health

administration departments should prioritize developing a supportive and innovative hospital culture that fosters positivity and collaboration, thereby enhancing healthcare professionals' motivation to participate, promoting MDT, and ultimately improving the quality of healthcare services.

5.6 The mediating role of participation motivation in the relationship between team leadership and MDT behavior

The bootstrap analysis demonstrated that participation motivation partially mediated the relationship between hospital culture and teamwork behavior in multidisciplinary settings. This aligns with previous study findings indicating that participation motivation mediates the relationship between transformational leadership and organizational learning culture [16]. Transformational leadership meets employees' psychological needs and enhances their intrinsic motivation, which in turn fosters continuous learning and encourages cooperative behavior among team members [16]. Rydenfält et al. [59] used the IPO model and found that leadership styles, as input factors, influenced cohesion and communication, which are key elements of collaboration. These factors, in turn, impacted team effectiveness and patient safety, which are the desired outcomes [59].

Furthermore, Aubé et al. [60] found that proactive participation in teams mediated the relationship between perceived shared understanding and team performance, with participation motivation serving as a mediator in this relationship. This study used the IPO model, categorizing team leadership as an input factor, participation motivation as a process factor, and healthcare professionals' MDT behavior as an output factor. The findings revealed that stronger team leadership was correlated with greater participation motivation among healthcare professionals, which in turn led to more positive MDT behavior. This highlights that healthcare professionals' participation motivation is

crucial for the effective functioning of MDT. Thus, hospital administrators should prioritize developing the leadership skills of their managers to boost healthcare professionals' participation motivation, foster MDT, improve teamwork efficiency, and ultimately enhance the quality of healthcare services.

5.7 Implications and limitations

This study introduced participation motivation as a mediating variable and developed a mediation model that links hospital culture, team leadership, and MDT behavior. This enriches the understanding of the factors influencing teamwork behavior in tertiary public hospitals. It clarifies the roles of hospital culture and team leadership in shaping MDT behavior through participation motivation and offers strategies to enhance collaborative behavior among team members. These insights provide a theoretical foundation for developing MDT in public hospitals, contributing to the establishment of a patient-centered healthcare delivery system based on effective teamwork.

This study had several limitations. First, due to the cross-sectional research design, this study could only infer correlations between variables and could not establish causal relationships. Future research should consider longitudinal or experimental designs to clarify the causal relationships between MDT behaviors and their influencing factors. Second, the study's sample was restricted to tertiary public hospitals in the economically developed city of Hangzhou, which may not reflect conditions in less economically developed regions, thus limiting the generalizability of the findings. Future studies should broaden the geographical scope by including survey samples from both eastern and western provinces and cities to enhance the representativeness of the results. Third, the detail of the research variables was inadequate. Future research could break down specific dimensions of hospital culture, team leadership, and

participation motivation, such as categorizing team leadership into trust, teamwork skills, conflict management, and crisis handling, to obtain deeper insights.

6. Conclusions

This study demonstrated that the culture of tertiary public hospitals in Hangzhou, effective team leadership, and the participation motivation of healthcare professionals significantly influenced MDT behavior. In particular, a positive hospital culture and strong team leadership significantly enhanced teamwork behavior in multidisciplinary settings. As the culture of the hospital and team leadership improve, an increase in teamwork behavior is anticipated. Moreover, participation motivation partially mediated the relationship between hospital culture, team leadership, and teamwork behavior.

Therefore, hospital management should prioritize fostering a positive hospital culture, enhancing team leadership, and boosting healthcare professionals' participation motivation through various incentives. By adopting comprehensive strategies, hospitals can enhance teamwork among healthcare professionals and establish patient-centered multidisciplinary diagnostic and treatment teams, ultimately improving the quality of healthcare services and advancing the development of public hospitals.

Acknowledgments

We would like to express our deepest gratitude to the Health Commission of Zhejiang Province for their support in conducting this study. We would also like to thank all study participants for their time. Moreover, we are grateful to Jixiang Lai, who helped improve this paper.

Competing interests

The authors declare no competing interests.

Data availability

The datasets generated during and/or analyzed during the current study are available from the corresponding author upon reasonable request.

Ethical approval

This study was approved on November 1, 2022, by the Ethics Review Committee of Hangzhou Normal University, including the study protocol, informed consent form, and questionnaire. All procedures were performed in accordance with the ethical standards of the responsible committee on human experimentation and the Declaration of Helsinki.

Funding

This research was funded by the National Natural Science Foundation of China Project (grant number 72274051) and Hangzhou Medicine and Health Science and Technology Program(grant number ZD20220099).

Author contributions

Conceptualization: XH; data curation: ZM; methodology: JT; software: JY and ZM; validation: XW and JT; formal analysis: JY; investigation: HW; writing—original draft preparation: ZM and XH; writing—review and editing: ZM and LC; visualization: ZM; supervision: XW. All authors have read and agreed to the published version of this manuscript.

Informed consent

Written informed consent was obtained from all participants before administering the questionnaire. Each participant was thoroughly informed about the study’s objectives, procedures, potential risks and benefits, and their right to confidentiality and anonymity. We assured them that their participation was voluntary and that they were free to decline to answer any question or withdraw from the study at any point without any negative repercussions.

References

1. Lin Z, Yunyue L, Di X, Jinping T. Research progress on the effectiveness of multidisciplinary team cooperation in geriatric medicine. Int. J. Geriatr. Med. 2023;44:751–754.

2. Linghe Y. Research on the current situation and model of multidisciplinary diagnosis and treatment in tertiary cancer hospitals based on embeddedness theory [dissertation]. Peking Union Medical College; 2023. Available from: 10.27648/d.cnki.gzxhu.2022.000587.

3. Tao L. Analysis of the implementation of multidisciplinary collaborative diagnosis and treatment model and countermeasures in a tertiary general hospital in Guangdong Province [dissertation]. Southern Medical Univ; 2017.

4. Li J, Zhengwei W, Guodong G, Jing H, Nilei X, Xiangdong Y. SWOT analysis of the implementation of multidisciplinary diagnosis and treatment model in public hospitals. Chin. Hosp. Manag. 2017;37:30–31.

5. Zeng Y, Zhu S, Wang Z, Chen J, Dai J, Liu Z, et al. Multidisciplinary team (MDT) discussion improves overall survival outcomes for metastatic renal cell carcinoma patients. J. Multidiscip. Healthc. 2023;16:503–513. doi: 10.2147/JMDH.S393457.

6. Jianzhong D, Kun L, Qinggui R, Yumei Y, Jie S, Bin G, et al. Research progress on the clinical application of multidisciplinary team diagnosis and treatment models. Chin. Hosp. 2016;20:79–80.

7. Qin L, Qian H, Xiaoli H, Linghong L, Huanlin W, Jialing D, et al. Application and practice of the multidisciplinary diagnosis and treatment service model at West China Hospital. West. Chin. Med. J. 2024;39:88–92.

8. Deneckere S, Euwema MI, Lodewijckx C, Panella M, Mutsvari T, Sermeus W, et al. Better interprofessional teamwork, higher level of organized care, and lower risk of burnout in acute health care teams using care pathways: A cluster randomized controlled trial. Med. Care. 2013;51:99–107. doi: 10.1097/MLR.0b013e3182763312.

9. Xiaoxi L, Wenhui T, Zhongxia W, Bo L, Jinghui D, Ying X, et al. Application of case teaching method in clinical teaching of infectious diseases under multidisciplinary collaboration model. Med. Educ. Manag. 2023;9:740–745.

10. Mcewan D, Ruissen GR, Eys MA, Zumbo BD, Beauchamp MR. The effectiveness of teamwork training on teamwork behaviors and team performance: A systematic review and meta-analysis of controlled interventions. PLOS ONE. 2017;12:e0169604. doi: 10.1371/journal.pone.0169604.

11. Listyowardojo TA, Yan X, Leyshon S, Ray-Sannerud B, Yu XY, Zheng K, et al. A safety culture assessment by mixed methods at a public maternity and infant hospital in China. J. Multidiscip. Healthc. 2017;10:253–262. doi: 10.2147/JMDH.S136943.

12. Veenstra GL, Dabekaussen KFAA, Molleman E, Heineman E, Welker GA. Health care professionals’ motivation, their behaviors, and the quality of hospital care: A mixed-methods systematic review. Health Care Manag. Rev. 2022;47:155–167. doi: 10.1097/HMR.0000000000000284.

13. Ellis LA, Tran Y, Pomare C, Long JC, Churruca K, Saba M, et al. Hospital organizational change: The importance of teamwork culture, communication, and change readiness. Front Public Health. 2023;11:1089252. doi: 10.3389/fpubh.2023.1089252.

14. Qiang L, Cong W, Jianfeng L. The relationship between multidisciplinary team collaboration behavior and organizational factors in tertiary public hospitals in Hangzhou City: A study based on a moderated mediation model. Chin. J. Health Policy. 2024;17:45–52.

15. Linnander E, McNatt Z, Boehmer K, Cherlin E, Bradley E, Curry L. Changing hospital organizational culture for improved patient outcomes: Developing and implementing the leadership saves lives intervention. BMJ Qual. Saf. 2021;30:475–483. doi: 10.1136/bmjqs-2019-010734.

16. Udin U. Transformational leadership and organizational learning culture in the health sector: The mediating and moderating role of intrinsic work motivation. Work. 2024;77:1125–1134. doi: 10.3233/WOR-230047.

17. Campion MA, Papper EM, Medsker GJ. Relations between work group characteristics and effectiveness: A replication and extension. Pers. Psychol. 1996;49:429–452. doi: 10.1111/j.1744-6570.1996.tb01806.x.

18. McGrath JE. Social psychology: A brief introduction. New York: Holt, Rinehart & Winston; 1964.

19. Hong T, Jingbo L, Laixin Z, En L. Research on the I-P-O relationship model of virtual research teams in general hospitals. Chin. Hosp. Manag. 2009;29:45–48.

20. Urbini F, Callea A, Chirumbolo A, Talamo A, Ingusci E, Ciavolino E. Team performance in the Italian NHS: The role of reflexivity. J. Health Organ. Manag. 2018;32:190–205. doi: 10.1108/JHOM-07-2017-0180.

21. Owens K, Eggers J, Keller S, McDonald A. The imperative of culture: A quantitative analysis of the impact of culture on workforce engagement, patient experience, physician engagement, value-based purchasing, and turnover. J. Healthc. Leadersh. 2017;9:25–31. doi: 10.2147/JHL.S126381.

22. Ballangrud R, Aase K, Vifladt A. Longitudinal team training programme in a Norwegian surgical ward: A qualitative study of nurses' and physicians' experiences with teamwork skills. BMJ Open. 2020;10:e035432. doi: 10.1136/bmjopen-2019-035432.

23. Qin X, Wang BL, Zhao J, Wu P, Liu T. Learn from the best hospitals: A comparison of the mission, vision, and values. BMC Health Serv. Res. 2023;23:792. doi: 10.1186/s12913-023-09699-8.

24. Zanon RE, Dalmolin GL, Magnago TS, Andolhe R, Carvalho RE. Presenteeism and safety culture: Evaluation of health workers in a teaching hospital. Rev. Bras. Enferm. 2021;74:e20190463. doi: 10.1590/0034-7167-2019-0463.

25. Xu D, Yaya W, Limin W, Jieru C, Weijiao Z, Shaomei S. Analysis of the current status and influencing factors of nurse-perceived medical-nurse cooperation in tertiary hospital departments. Chin. Nurs. Manag. 2019;19:1477–1481.

26. Real K, Bell S, Williams MV, Latham B, Talari P, Li J. Patient perceptions and real-time observations of bedside rounding team communication: The Interprofessional

Teamwork Innovation Model (ITIM). Jt. Comm. J. Qual. Patient Saf. 2020;46:400–409. doi: 10.1016/j.jcjq.2020.04.005.

27. Qingwen Z. Development and evaluation of an organizational performance-oriented corporate team leadership model [dissertation]. Tianjin Univ; 2018.

28. Graça AM, Passos AM. Team leadership across contexts: A qualitative study. Leadersh. Org. Dev. J. 2015;36:489–511. doi: 10.1108/LODJ-08-2013-0114.

29. Beiboer C, Andela R, Hafsteinsdóttir TB, Weldam S, Holtrop T, van der Cingel M. Teamwork, clinical leadership skills and environmental factors that influence missed nursing care: A qualitative study on hospital wards. Nurse Educ. Pract. 2023;68:103603. doi: 10.1016/j.nepr.2023.103603.

30. Akinsola GD, Farmanesh P, Mwamba NM. The relationship between knowledge risk management and sustainable organizational performance: The mediating and moderating role of leadership behavior. Econ. 2023;17:20220047. doi: 10.1515/econ-2022-0047.

31. Xu X, Jia S, Zhang S, Mai X, Mao Z, Han B. Analysis of the factors influencing teamwork among oncology nurses based on multigroup structural equation model. Ann. Transl. Med. 2022;10:1015. doi: 10.21037/atm-22-4217.

32. Tremblay D, Touati N, Roberge D, Denis JL, Turcotte A, Samson B. Conditions for production of interdisciplinary teamwork outcomes in oncology teams: Protocol for a realist evaluation. Implement. Sci. 2014;9:76. doi: 10.1186/1748-5908-9-76.

33. Yongchao Z. A study on the relationship between volunteer participation motivation, organizational commitment, and life satisfaction. Master's thesis, Nanjing Univ. of Science and Technology; 2019.

34. Cumbler E, Kneeland P, Hagman J. Motivation of participants in an interprofessional quality improvement leadership team. J. Interprof. Educ. Pract. 2016;3:5–7. doi: 10.1016/j.xjep.2016.03.005.

35. Yahang Y, Chen K, Wei Z, Beibei Y. A study on the serial mediating effect of work motivation on job satisfaction and job performance among primary health care personnel. Chin. J. Health Pol. 2021;14:21–28.

36. Yahang Y, Chen K, Beibei Y (2021). A systematic review of the impact of integrated health care system reform on the work motivation and behavior of medical staff. Chin J Health Policy 14:15–22

37. Chmielewska M, Stokwiszewski J, Filip J, Hermanowski T. Motivation factors affecting the job attitude of medical doctors and the organizational performance of public hospitals in Warsaw, Poland. BMC Health Serv. Res. 2020;20:701. doi: 10.1186/s12913-020-05573-z.

38. Huang X, Gao Y, Chen H, Zhang H, Zhang X. Hospital culture and healthcare workers' provision of patient-centered care: A moderated mediation analysis. Front. Pub. Health. 2022;10:919608. doi: 10.3389/fpubh.2022.919608.

39. Spies LA, Gray J, Opollo JG, Mbalinda S, Nabirye R, Asher CA. Transformational leadership as a framework for nurse education about hypertension in Uganda. Nurse Educ. Today. 2018;64:172–174. doi: 10.1016/j.nedt.2018.02.009.

40. Subramaniam C, Johari J, Mashi MS, Mohamad R. The influence of safety leadership on nurses' safety behavior: The mediating role of safety knowledge and motivation. J. Saf. Res. 2023;84:117–128. doi: 10.1016/j.jsr.2022.10.013.

41. Chaman S, Zulfiqar S, Shaheen S, Saleem S. Leadership styles and employee knowledge sharing: Exploring the mediating role of introjected motivation. PLOS ONE. 2021;16:e0257174. doi: 10.1371/journal.pone.0257174.

42. Tran YT, Hoang TC. Transformational leadership and public employee performance: The mediating roles of employee participation and public service motivation. Int. Rev. Admin. Sci. 2024;0.

43. Körner M, Wirtz MA, Bengel J, Göritz AS. Relationship of organizational culture, teamwork, and job satisfaction in interprofessional teams. BMC Health Serv. Res. 2015;15:243. doi: 10.1186/s12913-015-0888-y.

44. Jalil R, Soukup T, Akhter W, Sevdalis N, Green JS. Quality of leadership in multidisciplinary cancer tumor boards: Development and evaluation of a leadership assessment instrument (ATLAS). World J. Urol. 2018;36:1031–1038. doi: 10.1007/s00345-018-2255-1.

45. Tremblay MA, Blanchard CM, Taylor S, Pelletier LG, Villeneuve M. Work extrinsic and intrinsic motivation scale: Its value for organizational psychology research. Can. J. Behav. Sci. 2009;41:213–226. doi: 10.1037/a0015167.

46. Jin C, Xiangping C, Yuewen L, Yiyu Z. The Chinese version of the Simplified Interprofessional Teamwork Scale: Translation and psychometric analysis. Nurs. Rehab. 2019;18:1–6.

47. Lusi Y, Lin W, Dongmei L, Yufei W, Lu Z, Yanzhe C, et al. The relationship between nursing staff's teamwork, work ability, and team performance. Chin. Hosp. Manag. 2018;38:67–69.

48. Yutong T, Yan Z, Jing Z, Cccan D, Hongjie L, Zhen L. et al. Analysis of the readiness and influencing factors of undergraduate nursing students' interprofessional collaborative learning. Chin. J. Nurs. Educ. 2020;17:794–798.

49. Pavithra A, Westbrook J. An assessment of organizational culture in Australian hospitals using employee online reviews. PLOS ONE. 2022;17:e0274074. doi: 10.1371/journal.pone.0274074.

50. DiazGranados D, Dow AW, Appelbaum N, Mazmanian PE, Retchin SM. Interprofessional practice in different patient care settings: A qualitative exploration. J. Interprof. Care. 2018;32:151–159. doi: 10.1080/13561820.2017.1383886.

51. Mulvale G, Embrett M, Razavi SD. "Gearing up" to improve inter-professional collaboration in primary care: A systematic review and conceptual framework. BMC Fam. Pract. 2016;17:83. doi: 10.1186/s12875-016-0492-1.

52. Harun A, Kunjukunju A, Yusof P, Ahmad A. The effect of teamwork training through multidisciplinary approaches towards safety culture among operation theatre staff of a private hospital. Nur. Primary Care. 2020;4. doi: 10.33425/2639-9474.1163.

53. Chao Y, Jiamin J, Yunyun S, Dan Dan Z, Li Z, Chun Hua G. Application of the multidisciplinary team cooperation model in prone position ventilation therapy skill training in the ICU. Chin. J. Nurs. Educ. 2022;19:1011–1016.

54. Abdulrahman GO Jr. The effect of multidisciplinary team care on cancer management. Pan. Afr. Med. J. 2011;9:20. doi: 10.4314/pamj.v9i1.71195.

55. Wenhan Y, Tianyu J, Manli W. A study on the current status of tumor MDT operation management based on content analysis method. Chin. Hosp. Manag. 2020;40:50–53.

56. Latham GP, Winters DC, Locke EA. Cognitive and motivational effects of participation: A mediator study. J. Organ. Behav. 1994;15:49–63. doi: 10.1002/job.4030150106.

57. Karaferis D, Aletras V, Raikou M, Niakas D. Factors influencing motivation and work engagement of healthcare professionals. Mater. Sociomed. 2022;34:216–224. doi: 10.5455/msm.2022.34.216-224.

58. McFadden P, Henagan S, Gowen III CR. The association between health care staff engagement and patient safety outcomes: A systematic review and meta-analysis. J. Patient. Saf.

59. Rydenfält C, Odenrick P, Larsson PA. Organizing for teamwork in healthcare: An alternative to team training? J. Health Organ. Manag. 2017;31:347–362. doi: 10.1108/JHOM-12-2016-0233.

60. Aubé C, Rousseau V, Brunelle E, Marques D. The relevance of being “on the same page” to succeed as a project team: A moderated mediation model. Motiv. Emot. 2018;42:804–815. doi: 10.1007/s11031-018-9706-2.

Click here to access/download;Figure;Figures.docx

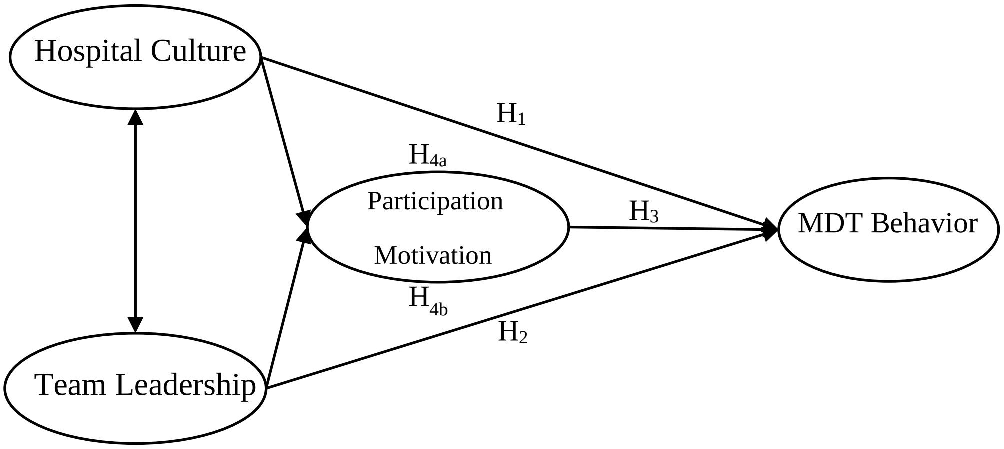

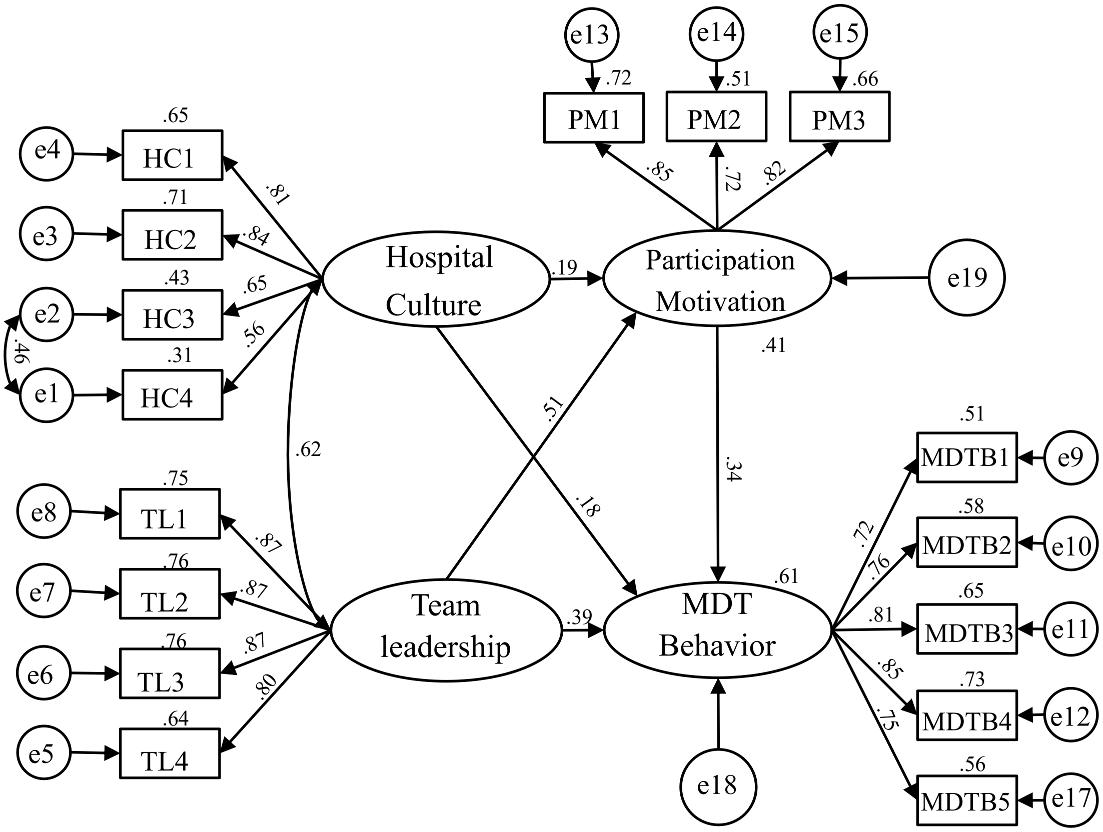

e13
e14
e15
.72
.51
.66
PM1
PM2
PM3
.85
.72
.82
e4
HC1
.65
e3
HC2
.71
e2
HC3
.43
e1
HC4
.31
.46
.81
.84
.65
.56
Hospital Culture
.19
Participation Motivation
e19
.41
.51
.18
.34
.62
e8
TL1
.75
e7
TL2
.76
e6
TL3
.76
e5
TL4
.64
.87
.87
.87
.80
Team leadership
.39
MDT Behavior
.61
e18
MDTB1
.51
e9
MDTB2
.58
e10
MDTB3
.65
e11
MDTB4
.73
e12
MDTB5
.56
e17
.72
.76
.81
.85
.75
