# Supplementary material for: Fostering participation motivation and multidisciplinary teamwork collaboration through hospital culture and team leadership in Chinese tertiary public hospitals—A cross-sectional study
Source: PLoS One. 2025 Nov 13;20(11):e0334831. doi: 10.1371/journal.pone.0334831 (PMC12614540; doi:10.1371/journal.pone.0334831)
Supplement: Attachment — Submitted filename: Response to Reviewers(8.30).docx [file pone.0334831.s005.docx]

August 30, 2025

Alejandro Botero Carvajal, Ph.D.

Academic Editor

*PLOS ONE*

**RE: “Fostering participation motivation and multidisciplinary teamwork collaboration through hospital culture and team leadership in Chinese tertiary public hospitals—a cross-sectional study”**

Dear Editor:

Thank you for the opportunity to further revise our manuscript “Fostering participation motivation and multidisciplinary teamwork collaboration through hospital culture and team leadership in Chinese tertiary public hospitals.” The reviewers’ and editors’ comments were useful and helped to clarify and improve several aspects of the manuscript.

Please find attached the revised version of the manuscript; the changes made are indicated using red font. Below is a point-by-point response to the issues raised by the reviewers and editors.

In addition, we would like to inform you of an update regarding the authors’ institutional affiliations. Due to a recent organizational restructuring at Hangzhou Normal University, the Department of Health Policy and Management, formerly part of the School of Public Health, was officially merged with the School of Public Administration on March 2, 2025. Accordingly, the affiliations of five authors —ZhengNan Meng, Jiamin Tang, Jie Yang, Xiaohe Wang, and Xianhong Huang — should be updated from the School of Public Health, Hangzhou Normal University, to the School of Public Administration, Hangzhou Normal University.

We hope that this version is suitable for publication in *PLOS ONE*. We look forward to hearing from you.

Xianhong Huang

School of Public Administration, Hangzhou Normal University

Hangzhou 311121, China

[hxh974291@163.com](mailto:hxh974291@163.com)

**Point-by-point response.**

**Editorial**

**Comment 1:**Please ensure that your manuscript meets PLOS ONE's style requirements, including those for file naming.

**Response:**Thank you for this important reminder. Following the *PLOS ONE* author guidelines, we have carefully revised the manuscript and all supplementary files to ensure full compliance with the journal’s style requirements. Specifically, we:

(a) Adjusted the manuscript formatting according to the *PLOS ONE* style template, including title page, abstract structure, tables, and figure captions.

(b) Standardized the reference list format strictly following *PLOS ONE* citation requirements.

(c) Renamed all files (main manuscript, figures, tables, and supporting information) according to *PLOS ONE*’s file naming conventions, ensuring consistency and clarity.

(d) Cross-checked all submission files to confirm that formatting and file names strictly meet the journal’s requirements.

These adjustments have fully addressed the style requirements, and the revised submission is now fully consistent with *PLOS ONE* standards.

**Comment 2:**In the ethics statement in the Methods, you have specified that verbal consent was obtained. Please provide additional details regarding how this consent was documented and witnessed, and state whether this was approved by the IRB.

**Response:**Thank you for this important query. The ethics statement has been revised in **[Methods](Page 12, Lines 275–286)** with the following clarifications:“Prior to data collection, all participants were verbally informed about the study objectives, procedures, confidentiality protections, voluntary participation, and their right to withdraw at any time without negative consequences. Verbal informed consent was obtained in the presence of a trained investigator, who served as a witness and immediately documented the consent in a standardized electronic log system. The log included the participant’s initials, date, and time of consent. All records were securely stored in an encrypted database accessible only to the research team, ensuring confidentiality and traceability. This verbal consent procedure, along with the study protocol and questionnaire, was reviewed and formally approved by the Ethics Committee of Hangzhou Normal University (Approval No. 2022-1121) prior to the initiation of the study.”

This revised description fully addresses the reviewer’s concern regarding documentation, witnessing, and IRB approval.

**Comment 3:**We note that the grant information you provided in the ‘Funding Information’ and ‘Financial Disclosure’ sections do not match.

**Response:**Thank you very much for your careful observation. The inconsistency between the “Funding Information” and “Financial Disclosure” sections was due to our oversight during manuscript preparation. We have carefully checked and revised the content to ensure that both sections now provide consistent and accurate information. The corrected statement is as follows: “This research was funded by the National Natural Science Foundation of China Project (grant numbers 72274051 and 71974050) and the Hangzhou Medicine and Health Science and Technology Program (grant number ZD20220099).” This statement is now identical in both the “Funding Information” and “Financial Disclosure” sections.

**Comment 4:**Thank you for stating the following financial disclosure:

“This research was funded by the National Natural Science Foundation of China

Project (grant numbers 72274051 and 71974050) and Hangzhou Medicine and Health

Science and Technology Program(grant number ZD20220099).”

Please state what role the funders took in the study. If the funders had no role,

please state. "The funders had no role in study design, data collection and analysis,

decision to publish, or preparation of the manuscript.”. If this statement is not correct

you must amend it as needed.

Please include this amended Role of Funder statement in your cover letter; we will

change the online submission form on your behalf.

**Response:**We appreciate this important clarification request. In accordance with *PLOS ONE*’s policy, we have explicitly stated the role of the funders. The following statement has been added both to the cover letter and to the manuscript to ensure consistency:“The funders had no role in study design, data collection and analysis, decision to publish, or preparation of the manuscript.” This correction ensures full compliance with *PLOS ONE*’s requirements.

**Comment 5:**Please note that funding information should not appear in the Acknowledgments section or other areas of your manuscript. We will only publish funding information present in the Funding Statement section of the online submission form. Please remove any funding-related text from the manuscript.

**Response:**Thank you for this important reminder. We have thoroughly reviewed the entire manuscript, including the Acknowledgments, main text, and footnotes, and have removed all funding-related information. The funding details are now presented only in the “Funding Statement” section of the online submission system. This revision ensures that the manuscript fully complies with *PLOS ONE’s* policy regarding the presentation of funding information.

**Comment 6:**We note that you have indicated that there are restrictions to data sharing for this study. For studies involving human research participant data or other sensitive data, we encourage authors to share de-identified or anonymized data. However, when data cannot be publicly shared for ethical reasons, we allow authors to make their data sets available upon request. For information on unacceptable data access restrictions, please see http://journals.plos.org/plosone/s/data-availability#loc-unacceptable-data-access-restrictions.

Before we proceed with your manuscript, please address the following prompts:

a) If there are ethical or legal restrictions on sharing a de-identified data set, please explain them in detail (e.g., data contain potentially identifying or sensitive patient information, data are owned by a third-party organization, etc.) and who has imposed them (e.g., a Research Ethics Committee or Institutional Review Board, etc.). Please also provide contact information for a data access committee, ethics committee, or other institutional body to which data requests may be sent.

b) If there are no restrictions, please upload the minimal anonymized dataset necessary to replicate your study findings to a stable, public repository and provide us with the relevant URLs, DOIs, or accession numbers. Please see http://www.bmj.com/content/340/bmj.c181.long for guidelines on how to de-identify and prepare clinical data for publication. For a list of recommended repositories, please see https://journals.plos.org/plosone/s/recommended-repositories. You also have the option of uploading the data as Supporting Information files, but we would recommend depositing data directly to a data repository if possible.

Please update your Data Availability statement in the submission form accordingly.

**Response:** Thank you for your guidance. In accordance with *PLOS ONE’s* data sharing policy, we have updated the Data Availability Statement as follows: “The datasets generated and analyzed during the current study contain potentially identifying information. Although direct identifiers (e.g., names, addresses) have been removed, the combination of narrow age bands, gender, professional title, and department codes from a limited number of tertiary hospitals may still allow for participant re-identification. As a result, the data cannot be made publicly available.

However, the data can be made available to qualified researchers who submit a formal request. To request access to the data, interested researchers should:

（1）Submit a written request to the Research Ethics Committee of Hangzhou Normal University at kejichu@hznu.edu.cn, clearly stating the purpose of their research and how the data will be used.

（2）Include a brief description of the research plan, specifying the type of data required.

（3）Agree to the terms and conditions set by the Ethics Committee for ensuring data privacy and security.

The Research Ethics Committee will evaluate each request based on ethical guidelines, the purpose of the research, and the potential risks of re-identification. If the request is approved, access to the data will be granted under strict confidentiality and security measures.”

**Comment 7:**In this instance it seems there may be acceptable restrictions in place that prevent the public sharing of your minimal data. However, in line with our goal of ensuring long-term data availability to all interested researchers, PLOS’ Data Policy states that authors cannot be the sole named individuals responsible for ensuring data access (http://journals.plos.org/plosone/s/data-availability#loc-acceptable-data-sharing-methods).

Data requests to a non-author institutional point of contact, such as a data access or ethics committee, helps guarantee long term stability and availability of data. Providing interested researchers with a durable point of contact ensures data will be accessible even if an author changes email addresses, institutions, or becomes unavailable to answer requests.

Before we proceed with your manuscript, please also provide non-author contact information (phone/email/hyperlink) for a data access committee, ethics committee, or other institutional body to which data requests may be sent. If no institutional body is available to respond to requests for your minimal data, please consider if there any institutional representatives who did not collaborate in the study, and are not listed as authors on the manuscript, who would be able to hold the data and respond to external requests for data access? If so, please provide their contact information (i.e., email address). Please also provide details on how you will ensure persistent or long-term data storage and availability.

**Response****:** We sincerely appreciate the journal's emphasis on safeguarding long-term

data availability for all interested researchers, which aligns with our commitment to

research transparency.

In accordance with *PLOS ONE's* data sharing policy, we understand the importance

of providing a non-author contact for long-term data availability. To comply with this

requirement, we have designated the Research Ethics Committee of Hangzhou Normal University as the permanent, independent point of contact for all future data requests. Interested researchers may reach the Committee at: [kejichu@hznu.edu.cn](mailto:kejichu@hznu.edu.cn). This institutional body is independent of the study team and can respond to data access inquiries should the corresponding authors become unavailable. Furthermore, to ensure the persistent storage and availability of the data, we will implement the following measures:

（1）All research data will be stored in the university’s research data management system, which complies with international standards for data storage and management, ensuring long-term preservation and stability.

（2）Data storage will adhere to the university’s data storage and backup policies, with regular backups and updates to mitigate risks from potential technical failures or unforeseen issues.

（3）The data will be registered on an appropriate platform and periodically updated to guarantee that external researchers can access the data, even if there are changes in the authorship.

We believe these measures will effectively ensure the long-term accessibility and transparency of the data, fully complying with *PLOS ONE's* requirements.

**Comment 8:**PLOS requires an ORCID iD for the corresponding author in Editorial Manager on papers submitted after December 6th, 2016. Please ensure that you have an ORCID iD and that it is validated in Editorial Manager. To do this, go to ‘Update my Information’ (in the upper left-hand corner of the main menu), and click on the Fetch/Validate link next to the ORCID field. This will take you to the ORCID site and allow you to create a new iD or authenticate a pre-existing iD in Editorial Manager.

**Response:**Thank you for your kind reminder. In accordance with the journal’s submission requirements, we have added and validated the corresponding author’s ORCID iD in the Editorial Manager system. The ORCID iD is: 0000-0002-5010-8082. This ensures full compliance with *PLOS ONE*’s policy on author identification and transparency.

**Comment 9:**Please amend the manuscript submission data (via Edit Submission) to include author “Simin Zhu”.

**Response:**Thank you for pointing this out. We apologize for the oversight. The name “Simin Zhu” should indeed be included as an author on this manuscript. We have now amended the submission record via Edit Submission to add this author and ensured that the author list is consistent across the manuscript, the submission system, and all related materials. All authors, including Simin Zhu, have confirmed the revised author list, which fully complies with the ICMJE authorship criteria and *PLOS ONE*’s authorship policies.

**Comment 10:**We note you have included a table to which you do not refer in the text of your manuscript. Please ensure that you refer to Table 4 in your text; if accepted, production will need this reference to link the reader to the Table.

**Response:**Thank you for your reminder. We have now added a clear reference to Table 4 in **[Results] (Page 19, Line 387)** when describing the corresponding findings, so that readers can easily locate and interpret the table. Additionally, we have carefully reviewed the entire manuscript to ensure that all tables are properly cited within the text. This revision ensures consistency between the text and tables, aligning with *PLOS ONE*’s formatting and production requirements.

**Comment 11:**Please review your reference list to ensure that it is complete and correct. If you have cited papers that have been retracted, please include the rationale for doing so in the manuscript text, or remove these references and replace them with relevant current references. Any changes to the reference list should be mentioned in the rebuttal letter that accompanies your revised manuscript. If you need to cite a retracted article, indicate the article’s retracted status in the References list and also include a citation and full reference for the retraction notice.

If the reviewer comments include a recommendation to cite specific previously published works, please review and evaluate these publications to determine whether they are relevant and should be cited. There is no requirement to cite these works unless the editor has indicated otherwise.

**Response:**Thank you for this important suggestion. We have carefully reviewed and cross-checked the entire reference list for completeness and accuracy, ensuring that it includes author names, publication years, journal titles, and DOIs. No retracted or withdrawn articles were identified. During this review, we made the following updates: corrected minor formatting errors in three references, updated two references to their most recent versions. We added one additional relevant article to strengthen the discussion. These revisions ensure that the reference list is complete, accurate, and fully consistent with *PLOS ONE*’s editorial standards.

**Response**

**To Reviewer 1:**

**Comment 1:**Study Design and Reporting Standards

The manuscript describes a cross-sectional study investigating healthcare professionals engaged in multidisciplinary teams.

The reporting should strictly adhere to STROBE Guidelines (Strengthening the Reporting of Observational Studies in Epidemiology)

Please ensure all 22 items in the STROBE checklist are adequately addressed in the manuscript.

**Response:**Thank you for this valuable suggestion. We have thoroughly revised the manuscript to ensure full compliance with the STROBE guidelines, and we have verified that all 22 checklist items are adequately addressed.

**（1）Title and abstract:** Explicitly state the cross-sectional design and provide a structured summary of objectives, methods, results, and conclusions.

**（2）Introduction:** Provides sufficient background on MDTs and specific study objectives.

**（3）Methods:** Expanded the description of study design, participant selection, variable definitions, measurement tools, sample size rationale, potential sources of bias, and detailed statistical methods.

**（4）Results:** Added clearer presentation of participant numbers at each stage and ensured complete reporting of descriptive and outcome data, supplemented by appropriate tables.

**（5）Discussion:** Strengthened the interpretation of findings in light of study limitations, potential biases, and generalizability, regarding prior evidence.

**（6）Other information:** In accordance with *PLOS ONE* policy and STROBE recommendations, funding information is reported in the Financial Disclosure section of the submission system.

A completed STROBE checklist (S2 Checklist), indicating the exact manuscript sections corresponding to each item, has been submitted as Supporting Information.

**Comment 2:** Data Quality and Management

Missing Data Analysis:

Quantify the extent of missing data (provide specific percentages)

Describe the patterns of missing data (completely at random, at random, or not at random)

Detail the statistical approaches used to handle missing data (e.g., complete case analysis, multiple imputation)

**Response****:**Thank you for your suggestion. In the revised manuscript **[Methods](Page 13–14, Lines 316–324)**, we have incorporated a detailed analysis of missing data: The proportion of missing values ranged from 1.1% to 2.9% across all study variables, indicating a low level of missingness. Little’s MCAR test indicated that the data were missing completely at random (MCAR) (P > 0.05) [1]. Given this low and random pattern of missing data, we applied multiple imputation using five imputed datasets. The imputation model was based on a multivariate normal regression approach with relevant auxiliary variables [2]. Rubin’s rule was used to pool estimates across datasets [3]. In addition, sensitivity analyses using complete case analysis yielded results consistent with the multiply imputed datasets, supporting the robustness of our findings.

***References***

1. Little RJA. A test of missing completely at random for multivariate data with missing values. J Am Stat Assoc. 1988;83(404):1198–1202. [doi:10.1080/01621459.1988.10478722](https://doi.org/10.1080/01621459.1988.10478722)

2. Collins LM, Schafer JL, Kam CM. A comparison of inclusive and restrictive strategies in modern missing data procedures. Psychol Methods. 2001 Dec;6(4):330–351. doi:10.1037/1082-989X.6.4.330

3. Rubin DB. Multiple Imputation for Nonresponse in Surveys. New York: John Wiley & Sons; 1987.

**Comment 3:**Data Transparency:

Include a clear statement about data availability

Specify the conditions and procedures for data access

**Response****:** Thank you for your suggestion. We have updated the Data Availability Statement to ensure clarity and transparency. The revised statement now reads: “The datasets generated and analyzed during the current study contain potentially identifying information. Although direct identifiers (e.g., names, addresses) have been removed, the combination of narrow age bands, gender, professional title, and department codes from a limited number of tertiary hospitals may still allow for participant re-identification. As a result, the data cannot be made publicly available.

However, the data can be made available to qualified researchers who submit a formal request. To request access to the data, interested researchers should:

（1）Submit a written request to the Research Ethics Committee of Hangzhou Normal University at kejichu@hznu.edu.cn, clearly stating the purpose of their research and how the data will be used.

（2）Include a brief description of the research plan, specifying the type of data required.

（3）Agree to the terms and conditions set by the Ethics Committee for ensuring data privacy and security.

The Research Ethics Committee will evaluate each request based on ethical guidelines, the purpose of the research, and the potential risks of re-identification. If the request is approved, access to the data will be granted under strict confidentiality and security measures.”

We hope this provides the necessary clarity regarding data availability and the procedures for accessing the data.

**Comment 4:**Sampling Methodology

Current Method: The study employs convenience sampling, please justify this sampling choice and acknowledge its limitations

Population Context: Provide the total estimated number of healthcare professionals in Hangzhou, include the breakdown of different healthcare professional categories if available, discuss how representative the sample is of the target population

**Response:**Thank you for the reviewer’s insightful comments regarding the sampling methodology.

**Justification for convenience sampling:** This study employed convenience sampling for several practical reasons:

(a) Exploratory research design: As an exploratory, cross-sectional survey, our study aimed to generate preliminary insights into multidisciplinary team (MDT) practices in tertiary public hospitals in Hangzhou. Convenience sampling is commonly adopted in exploratory health services research, as it facilitates rapid data collection and hypothesis generation [1,2].

(b) Lack of comprehensive sampling frame: No complete or up-to-date roster of MDT participants in tertiary hospitals is publicly available, making probability-based sampling infeasible.

(c) Resource constraints: Given limited time and funding, convenience sampling allowed us to recruit an adequate number of relevant respondents across multiple hospitals within a feasible timeframe.

**Limitations of convenience sampling:** While efficient, this approach may introduce selection bias and limit the generalizability of the results. In particular, the sample over-represents physicians compared to the general healthcare workforce, while nurses and medical technologists are under-represented. Consequently, the findings should be interpreted with caution and primarily in the context of MDT participants in tertiary hospitals, rather than generalized to all healthcare professionals in Hangzhou. This limitation has been explicitly acknowledged in the **[Discussion](Page 29–30, Lines 570–572).**

**Population Context:** According to the 2024 Hangzhou Statistical Yearbook [3], Hangzhou had a total of 161,189 healthcare professionals: 62,661 licensed (assistant) physicians (38.9%), 72,344 registered nurses (44.9%), 20,708 medical technologists (12.8%), and 5,846 other personnel (3.6%) in the overall data. In contrast, our sample included 452 MDT participants, as shown in Table 1; 79% were physicians, and 21% were nurses or technologists. This differs from the general workforce distribution but reflects the inherent composition of MDTs, which tend to include a higher proportion of senior physicians who lead interdisciplinary case discussions.

Despite these disparities, the study drew participants from seven tertiary public hospitals across multiple districts in Hangzhou. It covered MDTs from Internal Medicine, Surgery, Obstetrics & Gynecology, Pediatrics, and Medical Technology, thereby providing valuable insights into MDT practice in this context.

**Table 1. Composition of Healthcare Personnel in Hangzhou (2024 Statistical Yearbook data) vs. Study Sample**

| **Professional Title** | **Count (Proportion%)** | |
| --- | --- | --- |
|  | **Overall Data** | **Sample Data** |
| **Licensed (Assistant) Physician** | 62,661(38.9%) | 357(79%) |
| **Registered Nurse** | 72,344(44.9%) | 95(21%) |
| **Medical Technicians** | 20,708(12.8%) |  |
| **Others** | 5,846(3.6%) | 0(0%) |
| **Total** | **161,189(100%)** | **452(100%)** |

***References***

1. Etikan I, Musa SA, Alkassim RS. Comparison of convenience sampling and purposive sampling. Am J Theor Appl Stat. 2016;5(1):1–4. doi:10.11648/j.ajtas.20160501.11

2. Bornstein MH, Jager J, Putnick DL. Sampling in developmental science: Situations, shortcomings, solutions, and standards. Dev Rev. 2013;33(4):357–370. doi:10.1016/j.dr.2013.08.003

3. Hangzhou Municipal Bureau of Statistics. 2024 Hangzhou Statistical Yearbook. Hangzhou Municipal Bureau of Statistics website [Internet]. 2024-12-16 [cited 2025-08-10]. Available from: https://tjj.hangzhou.gov.cn/art/2024/12/16/art_1229453592_4320735.html

**To reviewer 2:**

**Comment 1:**The manuscript focus is on a topical and relevant concept today. The strengths of this paper is that it addresses the interplay of culture, participation motivation with MDT behavior as the outcome variable.

The concept of MDT and MDT behavior needs further elaboration to provide clarity as to what is the practice in the study area and what model is expected. The statistical analysis was rigorous. Overall, it was a good manuscript.

**Response:**Thank you for highlighting the need for conceptual clarity. We have expanded the definitions of both MDT and MDT behavior in the revised manuscript **[Introduction](Page 3, Lines 56-59)** and **[Methods](Page 11, Lines 261-262)** to provide a more explicit context specific to our study setting.

**Definition of MDT and MDT Behavior:** MDT refers to a structured and relatively stable group of three or more healthcare professionals from diverse specialties who meet regularly to conduct patient assessments, diagnosis, treatment planning, and follow-up [1]. MDT behavior refers to the observable patterns of communication, interaction, and collaboration among team members, with the shared goal of achieving optimal clinical outcomes [2].

**Context in the Study Setting:** In tertiary public hospitals in Hangzhou, MDTs are formally organized, with regular (weekly or biweekly) team meetings involving physicians, surgeons, nurses, and allied health staff. These MDTs follow protocol-based case discussions similar to those promoted by national MDT initiatives, such as those at major hospitals like the Children’s Hospital of Zhejiang University School of Medicine and Zhejiang Provincial People’s Hospital [3,4]. Therefore, the definition aligns well with MDT practice in our sampled institutions.

**Operationalization of MDT Behavior:** MDT behavior is operationalized through regular team meetings, interdisciplinary communication, and collaborative decision-making. These behaviors can be assessed through metrics such as team meeting frequency, communication efficiency, and interdisciplinary collaboration [5].

***References***

1. Taberna M, Gil Moncayo F, Jané-Salas E, Antonio M, Arribas L, Vilajosana E, et al. The multidisciplinary team (MDT) approach and quality of care. Front Oncol. 2020;10:85. Doi : 10.3389/fonc.2020.00085

2. Raine R, Wallace I, Nic a’Bháird C, Xanthopoulou P, Lanceley A, Clarke A, et al. Improving the effectiveness of multidisciplinary team meetings for patients with chronic diseases: a prospective observational study. Southampton (UK): NIHR Journals Library; 2014 Oct.

3. Children's Hospital of Zhejiang University School of Medicine. Multidisciplinary team (MDT) management of complex pediatric diseases at the Children's Hospital of Zhejiang University. Hangzhou News Center–Hangzhou Net [Internet].2022 Nov 17 [cited 2025 Aug 10]. Available from: <https://hznews.hangzhou.com.cn/kejiao/content/2022-11/17/content_8401827_0.htm>

4. Lin L, Yu QQ. Multidisciplinary diagnosis and treatment: advantages in managing complex diseases. City Express [Internet]. 2024 Jan 6 [cited 2025 Aug 10]. Available from: <https://hzdaily.hangzhou.com.cn/dskb/2024/01/06/article_detail_2_20240106A095.html>

5. Harris J, Taylor C, Sevdalis N, Jalil R, Green JSA. Development and testing of the cancer multidisciplinary team meeting observational tool (MDT-MOT). Int J Qual Health Care. 2016 Jul;28(3):332-8. doi:10.1093/intqhc/mzw030.

**to comments raised in the manuscript**

**Comment 1:**What constitutes modern disease should be defined as compared to ancient disease?

**Response:**Thank you for your valuable suggestion. In this study, modern diseases are defined as chronic, non-communicable conditions strongly linked to lifestyle and environmental factors, such as prolonged sedentary behavior, an imbalanced diet, and chronic stress [1]. These include cardiovascular disease, diabetes, mental health disorders, and cancer [2]. By contrast, ancient diseases mainly refer to historical infectious diseases such as cholera, plague, and smallpox. They were characterized by rapid transmission, short disease courses, and often single-department treatment approaches [3,4].

From a public health perspective, the key distinction lies in their nature: while ancient diseases were predominantly acute, infectious, and curable with targeted interventions, modern diseases are chronic, non-communicable, multifactorial, and require long-term management. Their complexity often involves multiple organ systems, thereby necessitating multidisciplinary teamwork and an integrated hospital culture to optimize patient outcomes.

***References***

1. Balwan WK, Kour S. Lifestyle diseases: the link between modern lifestyle and threat to public health. Saudi J Med Pharm Sci. 2021;7(8A):573-8.

2. Piovani D, Nikolopoulos GK, Bonovas S. Non-communicable diseases: the invisible epidemic. J Clin Med. 2022;11(19):5939. doi: 10.3390/jcm11195939

3. Hays JN. Epidemics and pandemics: their impacts on human history. Santa Barbara: ABC-CLIO; 2005.

4. World Health Organization. The World Health Report 1999: Making a Difference. Geneva: World Health Organization; 1999.

**Comment 2:**Same what makes a case complex?

**Response:**Thank you for raising this important point. In our study, complex cases refer to patients whose care cannot be adequately managed within a single specialty because of multiple overlapping factors. Specifically, these include:(a) multimorbidity involving two or more chronic conditions, (b) high diagnostic or therapeutic uncertainty, and (c) additional psychological, behavioral, or socio-economic challenges that affect treatment adherence and outcomes. This definition is consistent with the Vector Model of Complexity, which emphasizes the interplay among biological, social, cultural, environmental, and behavioral dimensions [1]. Consequently, complex cases necessitate collaborative decision-making and coordinated input from different specialties, which aligns directly with the focus of our study on multidisciplinary teamwork.

***References***

1. Safford MM, Allison JJ, Kiefe CI. Patient complexity: more than comorbidity. The vector model of complexity. J Gen Intern Med. 2007;22(Suppl 3):382-90. doi: 10.1007/s11606-007-0307-0

**Comment 3:**What constitutes MDT as opposed to inter-disciplinary care?

**Response:**Thank you for this critical question. To clarify, a multidisciplinary team (MDT) is defined as a relatively stable group of at least three clinicians from different specialties who independently assess a patient, formulate discipline-specific recommendations, and then share these through structured and regular meetings [1]. This model preserves clear professional boundaries and emphasizes coordination rather than integration. In contrast, interdisciplinary care (IDC) involves team members integrating their expertise across disciplinary lines to co-develop a unified care plan in real time, characterized by shared goals, shared decision-making, and continuous collaborative communication [2]. Figure 1 illustrates the conceptual and operational differences between MDT and IDC.

Fig 1. Comparison Between Multidisciplinary Team and Interdisciplinary Care

***References***

1. Taberna M, Gil Moncayo F, Jané-Salas E, Antonio M, Arribas L, Vilajosana E, et al. The Multidisciplinary Team (MDT) Approach and Quality of Care. Front Oncol. 2020 Mar 20;10:85. doi:10.3389/fonc.2020.00085.

2. Choi BC, Pak AW. Multidisciplinary, interdisciplinarity and transdisciplinary in health research, services, education and policy: 1. Definitions, objectives, and evidence of effectiveness. Clin Invest Med. 2006 Dec;29(6):351-64

**Comment 4:**Is there a standard of care for benchmarking MDT in a health facility?

**Response:**Thank you for raising this pivotal question. At present, there is no globally unified benchmark standard for multidisciplinary team (MDT) practice across healthcare institutions. However, several disease-specific guidelines and expert consensuses provide operational benchmarks that can be used to assess MDT quality.

For example, the UK NICE head-and-neck cancer guideline and the 2022 ACC/AHA/HFSA Heart Failure guideline both stipulate mandatory MDT management, with clearly defined core team members, meeting frequency, and documentation procedures [1,2]. Similarly, in China, the Expert Consensus on Perioperative Brain Health in the Elderly and the Chinese Expert Consensus on Multidisciplinary Diagnosis and Treatment of Lung Cancer provide structured protocols for team composition, activation criteria, and decision-making processes [3,4].

While these frameworks provide detailed guidance on team composition, responsibilities, and operational procedures, the implementation of MDTs varies across regions due to differences in healthcare systems, resources, and cultural contexts. Despite these variations, the widespread adoption of MDTs as a cornerstone of high-quality patient care underscores the importance of ongoing research and refinement of these practices.

***References***

1.Homer JJ, Winter SC, Abbey EC, Aga H, Agrawal R, ap Dafydd D, et al. Head and Neck Cancer: United Kingdom National Multidisciplinary Guidelines, Sixth Edition. The Journal of Laryngology & Otology. 2024;138(S1):S1–224. doi:10.1017/S0022215123001615

2.Sokos, G., Kido, K., Panjrath, G., Benton, E., Page, R., 2nd, Patel, J., Smith, P. J., Korous, S., & Guglin, M. (2023). Multidisciplinary Care in Heart Failure Services. Journal of cardiac failure, 29(6), 943–958. https://doi.org/10.1016/j.cardfail.2023.02.011

3.Wang, T. L., & Wang, D. X. (2019). Zhonghua yi xue za zhi, 99(27), 2081–2083. <https://doi.org/10.3760/cma.j.issn.0376-2491.2019.27.001>

3.Zhong W, Chinese Thoracic Oncology Research Group, Lung Cancer Committee of China Anti-Cancer Association, et al. Chinese expert consensus on multidisciplinary team diagnosis and treatment of lung cancer. Zhonghua Zhong LiuZa Zhi. 2020;42(10):817-828. doi:10.3760/cma.j.cn112152-20200812-00731

**Comment 5:**Was sample size computed? Or what was the power of the study?

**Response****:**Thank you for raising this important question. Regarding sample-size calculation, we followed Thompson’s SEM guideline: the ratio of observed variables to respondents should be at least 10:1, with 15:1–20:1 considered optimal [1]. The present study comprises 16 observed variables; assuming a conservative figure of 20 cases per variable yields a total of 320 respondents. Anticipating incomplete or inaccurate responses, we inflated this figure by an additional 20% to 384. From January 10, 2023, to July 20, 2023, we distributed 500 questionnaires and obtained 452 valid responses, corresponding to an effective response rate of 90.4%. To ensure adequate statistical power, a post hoc power analysis was conducted using G*Power 3.1 to assess the adequacy of the achieved sample size (6 predictors, f² = 0.15, α = 0.05, N = 452). The achieved statistical power (1 – β) was 0.99 [2]. This finding indicates that the study had more than sufficient power to detect medium-sized effects, thereby supporting the robustness of the statistical inference [3]. We will add these details to **[Methods](Page 9, Lines 205-216)** of the revised manuscript.

***References***

1. Thompson B. The ten commandments of good structural equation modeling behavior: a user-friendly, introductory primer on SEM. In: Proceedings of the Annual Meeting of the U.S. Department of Education, Office of Special Education Programs (OSEP) Project Directors’ Conference; 1998 Jul 16; Washington, DC.\

2. Faul F, Erdfelder E, Lang A-G, Buchner A. G*Power 3: A flexible statistical power analysis program for the social, behavioral, and biomedical sciences. Behav Res Methods. 2007;39(2):175-191. doi:10.3758/BF03193146.

3. Wolf EJ, Harrington KM, Clark SL, Miller MW. Sample size requirements for structural equation models: an evaluation of power, bias, and solution propriety. Educ Psychol Meas. 2013;76(6):913-34. doi: 10.1177/0013164413495237

**Comment 6:**How was the composite indices calculated? Responses that were Likert scale responses? Were they computed as means for analysis? How does this affect validity?

**Response:**Thank you for this helpful question. In our study, each composite index was calculated as the simple arithmetic mean of the items on its Likert scale (range: 1-5), which is the sum of item scores divided by the number of items. To address concerns regarding validity, we conducted reliability and validity analyses to ensure the accuracy of our results. All composite scales showed satisfactory internal consistency (Cronbach’s α values ranged from 0.739 to 0.913), and confirmatory factor analysis (CFA) indicated adequate structural validity, with all standardized factor loadings exceeding 0.50 and AVE values >0.50. These findings suggest that averaging items did not compromise the validity of the constructs. This clarification has been added to the revised manuscript **[Methods](Page 10-11, Lines 231-232；242-244；254-256；265-267).**

**Comment 7:**A description of the research tool should be done to explain the research variables - this is necessary to unpack the measures.

**Response:**Thank you for this helpful suggestion. In the revised manuscript, we have provided a more detailed description of the research tool and the operationalization of all study variables in **[Methods](Page 10–11, Lines 226–229; 237–240; 249–252; 261–264).** Specifically, for each construct (e.g., hospital culture, team leadership, participation motivation, and multidisciplinary collaboration), we now report: (i) the measurement source or basis (adapted from validated scales or self-developed items), (ii) the number of items and response format, and (iii) sample items for illustration. We also report the reliability and validity results (Cronbach’s α, CFA loadings, AVE/CR), confirming that all measures demonstrated acceptable psychometric properties. To ensure transparency and replicability, the complete English questionnaire has been uploaded as Supporting Information (S1 Appendix), allowing reviewers and readers full access to the instrument.
